# Development and validation of search filters to identify articles on deprescribing in Medline and Embase

**DOI:** 10.1186/s12874-022-01515-x

**Published:** 2022-03-25

**Authors:** Thomas Morel, Jérôme Nguyen-Soenen, Wade Thompson, Jean-Pascal Fournier

**Affiliations:** 1 Département de Médecine Générale, Faculté de Médecine, Nantes Université, Nantes, France; 2 Inserm-University of Tours-University of Nantes, UMR U1246 Sphere “Methods in Patient-Centered Outcomes and Health Research”, 37000 Tours, France; 3 Women’s College Hospital Research Institute, Toronto, ON Canada; 4 Research Unit of General Practice, University of Southern Denmark, Odense, Denmark

**Keywords:** Deprescriptions, Systematic review, Methods, Medical subject headings, Sensitivity

## Abstract

**Background:**

Deprescribing literature has been increasing rapidly. Our aim was to develop and validate search filters to identify articles on deprescribing in Medline via PubMed and in Embase via Embase.com.

**Methods:**

Articles published from 2011 to 2020 in a core set of eight journals (covering fields of interest for deprescribing, such as geriatrics, pharmacology and primary care) formed a reference set. Each article was screened independently in duplicate and classified as relevant or non-relevant to deprescribing. Relevant terms were identified by term frequency analysis in a 70% subset of the reference set. Selected title and abstract terms, MeSH terms and Emtree terms were combined to develop two highly sensitive filters for Medline via Pubmed and Embase via Embase.com. The filters were validated against the remaining 30% of the reference set. Sensitivity, specificity and precision were calculated with their 95% confidence intervals (95% CI).

**Results:**

A total of 23,741 articles were aggregated in the reference set, and 224 were classified as relevant to deprescribing. A total of 34 terms and 4 MeSH terms were identified to develop the Medline search filter. A total of 27 terms and 6 Emtree terms were identified to develop the Embase search filter. The sensitivity was 92% (95% CI: 83–97%) in Medline via Pubmed and 91% (95% CI: 82–96%) in Embase via Embase.com.

**Conclusions:**

These are the first deprescribing search filters that have been developed objectively and validated. These filters can be used in search strategies for future deprescribing reviews. Further prospective studies are needed to assess their effectiveness and efficiency when used in systematic reviews.

**Supplementary Information:**

The online version contains supplementary material available at 10.1186/s12874-022-01515-x.

## Introduction

Polypharmacy and inappropriate prescribing represent a major public health issue as the population is aging [1]. Their impact on morbidity and mortality is well-documented [2]. Deprescribing is the process of tapering or withdrawing inappropriate medications under the supervision of a health care professional [3]. Deprescribing is a positive patient-centered intervention which is part of the good prescribing continuum [1]. Identification of inappropriate medications and their careful, monitored discontinuation are key concepts for deprescribing. The concept of deprescribing emerged in 2000’s, and Reeve et al. proposed a first literature-based definition of deprescribing in 2015 [4]. Deprescribing literature has been increasing rapidly, including high-quality evidence and guidelines for health care professionals (e.g. deprescribing of chronic proton pump inhibitors [5] or benzodiazepine receptor agonists) [6].

Systematic reviews aim to summarize the evidence on a specific research question by retrieving all relevant articles using a carefully developed search strategy [7]. The Cochrane collaboration has proposed a guideline to develop search strategies for systematic reviews [8]. A search filter is a tool designed to improve effectiveness of a literature search in a specific database (e.g. Medline, Embase) [9]. Search filters are combined to search strategy to retrieve studies with a specific design (e.g. The Cochrane High Sensitive Search Strategy to identify randomized trials) or studies on a specific topic (e.g. High-Sensitivity Chronic Kidney Disease Search Filters for PubMed) [8, 10].

The process of developing a search filter includes evaluating its performance. Performance of a search filter reflects the capacity of the filter to retrieve relevant articles (sensitivity) and to exclude non-relevant articles (specificity and precision) [11]. Sensitivity, i.e. the proportion of relevant articles retrieved, is the most critical performance measure for systematic reviews [11]. Precision, i.e. the proportion of retrieved articles that are relevant, and specificity, i.e. the proportion of non-relevant articles not retrieved, is also essential to estimate the efficiency improvement of the search filter [11]. Some filters are developed with the aim to maximize sensitivity at the expense of a lower precision [12].

The performance of a search filter is measured against a reference set of relevant and non-relevant database-specific articles [13]. The validation of a search filter refers to exploring its performance on a reference set of relevant articles that have not been used to develop the filter [14].

To our knowledge, no search filter focused on deprescribing have been developed to date. Authors have used various title and abstract terms (free-text terms) to identify deprescribing articles when conducting systematic reviews (e.g. withdrawal, cessation, discontinuation). The extent to which such approaches can capture all relevant deprescribing literature is unclear. Subject headings were only created in 2016 with “Deprescriptions” as a Medical Subject Heading (MeSH) for Medline, and “Deprescription” as a Embase Subject Heading (Emtree). An optimal search strategy to retrieve deprescribing literature would include both free-text terms and subject headings identified as relevant to deprescribing. With deprescribing literature rapidly increasing, there is a need for a performant and validated deprescribing search filter to overcome indexing and vocabulary pitfalls and to ensure reviews are comprehensive in capturing all available literature. A deprescribing search filter should be considered as a topic filter, aiming to encompass the scope of this complex concept [9]. The aim of this study was to develop and validate a deprescribing search filter for Medline via PubMed and Embase via Embase.com maximizing sensitivity.

## Methods

### Study design

The study involved a four-step process:

1. We created a reference set of articles identified as relevant and non-relevant to deprescribing. The reference set was randomly divided into a development set (70% of the articles) and a validation set (30% of the articles).
2. We identified free-text terms and subject heading relevant to deprescribing using term frequency analysis in the development set.
3. We developed deprescribing search filters with maximized sensitivity for PubMed and Embase.com using the development set.
4. We evaluated the performances of the search filters using the validation set.

### Creation of the reference set

We selected a core set of eight journals on the basis of the opinions of international experts on deprescribing. Deprescribing experts were those who have regularly published in the deprescribing field, have led seminal publications on deprescribing and continue to be involved in international deprescribing research. These experts were convenient direct contacts of the authors. The selected journals had regularly published deprescribing articles from 2011 to 2020. We selected this period to take into account articles published before Reeve et al.’s definition and the creation of MeSH and Emtree subject heading [4]. This methodological approach to create the reference set was suggested by Lefebvre et al. [11].

We calculated that 139 articles would be necessary to a filter validate with a sensitivity of 90%. with a confidence interval of 95% (95% CI) ±5%. A validated filter with a 90% sensitivity was considered acceptable. Preliminary data suggested a prevalence of 2% for deprescribing articles in this set of journals. This estimated prevalence was considered sufficient to retrieve enough articles to develop and validate the search filters.

We included all published articles indexed both in Medline and Embase, so we could develop filters for PubMed and for Embase.com using the same dataset. Duplicates were excluded. Remaining articles constituted our reference set that was exported to the RAYYAN software for review [15].

### Article review

Three authors (TM, JNS, JPF) reviewed titles and abstracts for all articles in duplicate. Reviewers were blinded to each other’s judgements throughout the process. Articles were classified as relevant or non-relevant to deprescribing. Full copies were obtained for all articles with insufficient details. Any discrepancies were discussed and resolved by consensus with the third author. In case of persistent discrepancy, a fourth author (WT) was contacted to obtain consensus.

We defined inclusion criteria for articles relevant to deprescribing on the basis of the definition of Reeve et al. [4], and using input from international experts. Articles were included as relevant to deprescribing if they matched the following definition:

1. the intervention was designed with the goal of stopping or reducing the dose of an inappropriate medication
2. inappropriate medications were long-term medication
3. the intervention was supervised by a health care professional
4. or the intervention was performed according to a protocol designed by a health care team.

Inappropriate medications were defined as those where the risks outweigh the benefit. Long-term was defined by more than 4 weeks of use. Protocols designed by a health care team could include tools to guide the intervention (e.g. use of potentially inappropriate medications list before the initiation of the deprescribing intervention). Articles were included regardless of the type of reporting (original articles, reviews, editorials, commentaries, etc.), the study design, the study population, the nature of comparator (when appropriate) or the outcome used.

Articles were classified as non-relevant to deprescribing if they were focused on:

1. the misuse of a medication,
2. the effect of discontinuation of a medication with no deprescribing intervention,
3. the patient-initiated discontinuation without supervision of a health care professional (adherence issue).

We then randomly divided the reference set in a development set (70% of the reference set) and a validation set (30% of the reference set). Randomization was stratified on articles relevance, so the development set included 70% of the relevant to deprescribing articles and 70% of the non-relevant to deprescribing articles.

### Term frequency analysis

We adapted the strategy developed by Hausner et al. for search term identification [16]. We chose to follow this “objective” strategy for reproducibility. In this context, “objective” and “subjective” refer to the processes of term identification. In a “subjective” process, terms are usually identified by exploring thesaurus and questioning librarians and health care professional experts on the topic. Hausner et al. stated that the advantages of an “objective” approach are clearly described and reproductible methods to identify terms, whereas the selection of terms using a search strategy using a “subjective” approach remains partial and/or unclear [16].

We used the development set to identify the relevant search terms and to develop the filters. We performed a term frequency analysis on free-text terms, i.e. terms used on title and abstract, and Emtree subject headings using the Text Mining package in R software (version 4.0.3). Free-text terms identified in 10% or more of relevant articles were analyzed with Antconc software [17]. This software allowed a contextual analysis for each term. We identified truncations, phrases and terms colocation that were relevant for deprescribing. We calculated the frequency with which the terms appeared in the development set. Generic terms, terms that referred to a specific population, a study setting, or a study design were excluded, since they could bias the filter by focusing it inappropriately on a specific population or a specific study design. A generic term was a single word with a definition too broad to be related to the deprescribing concept when use outside a phrase. MeSH terms were identified and analyzed with PubReminer software [18].

### Development of deprescribing search filters

Deprescribing filters for Medline via PubMed and Embase via Embase.com were developed by an incremental approach, aiming at maximizing sensitivity [19]. We first used terms identified in 20% or more of relevant articles and 2% or less of non-relevant articles (i.e. these terms were both sensitive and specific to deprescribing). We selected the term with the highest frequency and then tested the term in combination with each of other terms. We calculated the incremental increase in sensitivity for each combination. We then selected the most sensitive combination and combined it with each of remaining terms. The process was continued until we could not increase the sensitivity by combining more terms. We then examined relevant articles from the development set that had not been retrieved by this filter and used terms identified in 10% or more of relevant articles or 3% or less of non-relevant articles to improve sensitivity using the same process. If relevant articles were still being missed after this step, we added specific search terms relevant to deprescribing but that were identified in less than 10% of relevant articles in order to improve sensitivity. We stopped developing the search filter when sensitivity could not be improved anymore with search terms relevant to deprescribing. We documented all search lines generated during the process, as well as the number of records retrieved in the development set for each search lines. At the end of this process, we created two filters (one for PubMed, one for Embase.com) with the highest sensitivity in the development set.

### Validation of deprescribing search filters

We applied each filter on the validation set and documented sensitivity, specificity and precision in the validation set, with their 95% CI, as suggested by Lefebvre et al. [11]. We documented which relevant articles from the validation set were missed.

### Secondary analyses

We assessed capacity of each filter for retrieving original deprescribing articles in the validation set. Original deprescribing articles were articles that reported original scientific research. Such articles were expected to be included in a systematic review. Review, editorials, letters and notes were excluded. We documented sensitivity for original articles in the validation set, with their 95% CI.

## Results

### Reference set

**Fig. 1:**
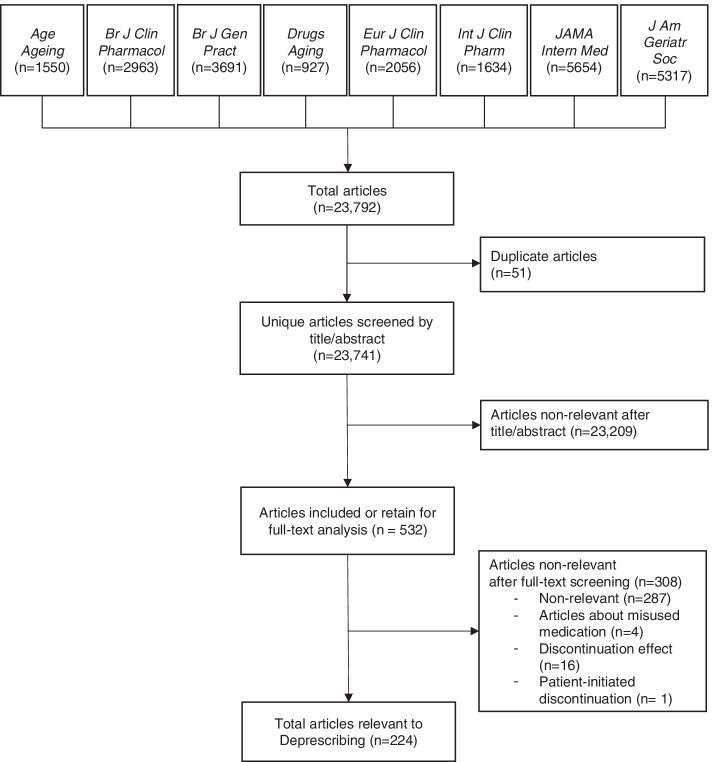
Flowchart for the creation of the reference set

Between 2011 and 2020, the eight journals published 23,792 articles indexed both in Embase and Medline that were eligible for the reference set. At the end of the screening process, 224 articles were classified as relevant to deprescribing, and 23,517 as non-relevant to deprescribing (Fig. 1).

Characteristics of relevant and non-relevant articles are described in Table 1. Among relevant articles, 55 were published during the 2011–2015 period and 169 during the 2016–2020 period. *Drugs & Aging* published 48 (21.4%) relevant articles but contributed to 867 (3.7%) of the non-relevant articles. Among the relevant articles in the reference set, 132 (58.9%) articles were original studies, 50 (22.3%) were reviews and 42 (18.8%) were commentaries, editorials or letters.

**Table 1:** Characteristics of the reference set

	Non-relevant articles *n* = 23,517	Relevant articles *n* = 224
Journals (%)		
*Age and Ageing*	1535 (6.5)	15 (6.7)
*British Journal of Clinical Pharmacology*	2940 (12.5)	23 (10.3)
*British Journal of General Practive*	3667 (15.6)	23 (10.3)
*Drugs and Aging*	866 (3.7)	48 (21.4)
*European Journal of Clinical Pharmacology*	2039 (8.7)	17 (7.6)
*International Journal of Clinical Pharmacy*	1599 (6.8)	31 (13.8)
*JAMA Internal Medicine*	5605 (19.6)	21 (8.5)
*Incl. Archives of Internal Medicine (2011–2012)*	991 (4.2)	2 (0.9)
*Journal of the American Geriatrics Society*	5266 (22.4)	46 (20.5)
Publication year (%)		
2011–2015	11,306 (48.1)	55 (24.6)
2016–2020	12,211 (51.9)	169 (75.4)
Publication type (%)		
Article	12,647 (53.8)	132 (58.9)
Review	1693 (7.2)	50 (22.3)
Other type^a^	9177 (39.0)	42 (18.8)

^a^Editorials, Letters, Notes, Short Survey, Tombstone

*Incl.* including

We randomly assigned 149 relevant articles and 15,678 non-relevant articles to the development set, and 75 relevant articles and 7839 non-relevant articles to the validation set.

### Medline search filter development

We identified 201 unique search terms identified in more than 10% of relevant articles. We identified 41 truncated terms or phrases using Antconc. From these 242 terms, 180 were excluded. Among excluded terms, 96 were generic terms, 49 were methodological terms, 15 were population-related terms and 20 were setting-related terms.

From the 62 remaining terms, 34 were identified to develop the filter. Among MeSH terms, 4 were identified to develop the filter. The terms and MeSH terms are presented in Supplementary Table S1.

At the end of the development process, a search filter was created using a combination of 18 terms, MeSH terms, phrases or truncated terms (Table 2) and retrieved 143 of the 149 relevant records (sensitivity: 96%) and 1583 of the non-relevant 15,678 (precision: 8%) of the development set. We added the term “taper*” and the phrase “dose reduction” at the end of the process since they were related to deprescribing concept and increased sensitivity, though they were identified in less than 10% of relevant articles. The analysis of the six missed articles did not lead to the identification of any supplementary relevant terms.

**Table 2:** Deprescribing search filter for Medline via PubMed

"deprescrib*"[Title/Abstract] OR "deprescriptions"[MeSH Terms] OR (("medication*"[Title/Abstract] OR "prescribing"[Title/Abstract]) AND "inappropriate"[Title/Abstract]) OR "polypharmacy"[Title/Abstract] OR "discontinu*"[Title/Abstract] OR ("withdraw*"[Title/Abstract] AND "medication*"[Title/Abstract]) OR (("medication*"[Title/Abstract] OR "drugs"[Title/Abstract] OR "prescribing"[Title/Abstract] OR "inappropriate"[Title/Abstract]) AND "reduc*"[Title/Abstract]) OR "inappropriate prescribing"[MeSH Terms] OR ("review*"[Title/Abstract] AND "medication"[Title/Abstract]) OR ("dose reduction"[Title/Abstract] OR "taper*"[Title/Abstract])	

### Medline search filter validation

The Medline search filter retrieved 69 of 75 relevant articles of the validation set and 777 of the 7839 non-relevant articles. The sensitivity was 92% (95% CI: 83–97), the precision was 8% (95% CI: 6–10%) and the specificity was 90% (95% CI: 89–91). Among the articles that were not retrieved, there was one original articles, one editorial and two letters and two notes. Performances are presented in Table 3.

**Table 3:** Performances of the search filters in the validation set

Search Filters	Relevant records retrieved in the validation set (***n*** = 75)	Non-relevant articles retrieved in the validation set (***n*** = 7839)	Sensitivity, % (95% CI)	Specificity, % (95% CI)	Precision, % (95% CI)	Number of articles retrieved in the database^**a**^
**Embase Filter**	68	771	91 (82–96)	90 (89–91)	8 (6–10)	635,289
**Medline Filter**	69	777	92 (83–97)	90 (89–91)	8 (6–10)	440,575

^a^ On June 16, 2021

*CI* Confidence Interval

The Medline search filter identified 35 of 36 original articles of the validation set. The sensitivity was 97% (95% CI: 85–100). The only original article missed by the filter was an expert opinion paper from the American Geriatrics Society published in 2012 that had no abstract [20].

### Embase search filter development

We identified 197 unique search terms identified in more than 10% of relevant articles. We identified 46 truncated terms or phrases using Antconc. From these 243 terms, 176 were excluded. Among excluded terms, 93 were generic terms, 49 were methodological terms, 15 were population-related terms and 19 were setting-related terms.

From the 67 remaining terms, phrases or truncated terms, 27 were identified to develop the filter. Among Emtree terms, 6 were identified to develop the filter. The terms and Emtree terms are presented in Supplementary Table S2.

At the end of the development process, a search filter (Table 4) which retrieved 144 of the 149 relevant records (sensitivity: 97%) and 1474 of the 15,678 non-relevant records (precision: 9%) was achieved using a combination of 18 terms, Emtree terms, phrases or truncated terms. As we did with the Medline search filter, we added the term “taper*” and the phrase “dose reduction” to the Emtree filter. The Emtree Terms “Deprescription” and “Inappropriate prescribing” were not used first during the process, since they did not improve the sensitivity of the search filter. However, we decided to combine them in the filter since they are critical for the deprescribing concept. The analysis of the five missed articles did not lead to the identification of any supplementary relevant terms.

**Table 4:** Deprescribing search filter for Embase via Embase.com

‘deprescrib*’:ab,ti OR ((review* NEAR/3 medication*):ab,ti) OR (((medication* OR medicines OR prescribing) NEAR/4 inappropriate):ab,ti) OR ‘potentially inappropriate’:ab,ti OR ((reduc* NEAR/5 medication*):ab,ti) OR ‘polypharmacy’:ab,ti OR ‘discontinu*’:ab,ti OR ‘withdraw*’:ab,ti OR ((reducing NEAR/1 (drug* OR inappropriate OR frid)):ab,ti) OR ‘polypharmacy’/de OR ‘medication therapy management’/de OR ‘dose reduction’:ab,ti OR ‘taper*’:ab,ti OR ‘drug withdrawal’/de OR ‘deprescription’/de OR ‘inappropriate prescribing’/de	

### Embase search filter validation

The Embase search filter retrieved 68 of the 75 relevant articles of the validation set and 771 of the 7839 non-relevant articles. The sensitivity was 91% (95% CI: 82–96), the precision was 8% (95% CI: 6–10) and the specificity was 90% (95% CI: 89–91). Among the articles that were not retrieved, there were five original articles, one review and one letter. Performances are presented in Table 3.

The Embase search filter identified 31 of 36 original articles of the validation set. The sensitivity was 86% (95% CI: 71–95) for identifying original articles. Among original articles that were not retrieved, 4 were published before 2016.

Overall, two articles were not retrieved by any of the two filters: an expert opinion paper published in 2012 [20] and a letter published in 2013 [21]. Both were published in the Journal of the American Geriatrics Society, and none had abstract available.

## Discussion

We developed two filters to identify articles on deprescribing for Medline and Embase with an estimated sensitivity of 92 and 91% respectively. To our knowledge, these are the first validated search filters for deprescribing articles in Medline and Embase. These search filters will be useful to researchers conducting deprescribing reviews, to ensure reviews are comprehensive in capturing all relevant literature.

By using an “objective” methodology [22] to identify relevant terms, our study provides an update on the vocabulary used in deprescribing literature. Many terms are directly connected to deprescribing (“inappropriate medication”, “discontinuation”, etc.). Other terms were more frequent than expected. The term “medication review*” was present in nearly 20% of relevant articles, highlighting that many deprescribing interventions are nested into medication review processes. Retrieving these articles in a systematic review process is key to ensure exhaustivity.

Furthermore, the result of frequency analysis showed that indexing of deprescribing articles with specific deprescribing subject headings is low. One out of three of the relevant articles were indexed with the MeSH term “Deprescriptions” in Medline and one out of five with the Emtree subject heading “Deprescription” in Embase. Several reasons may explain this finding. First, these subject headings were created recently and thus miss articles published before 2016. However, 88 of relevant articles in our reference set published after 2015 used the term “deprescribing” and 19 of them did not use the MeSH term “Deprescriptions”. Second, the definition proposed for the MeSH term did not perfectly match deprescribing definition, since the notion of inappropriate medication was not clearly mentioned. Hence, discontinuation articles could also be indexed under this MeSH term. As Gnjidic and Reeve suggested, medication discontinuation trials should be distinguished from deprescribing trials [3]. In medication discontinuation trials, discontinuation is attempted in all participants in the intervention group in order to explore benefice and harm of the discontinuation. Medication inappropriateness is related to the population group to which the participants belong, whereas in deprescribing trials medication inappropriateness is related to the personal medical context of each targeted participants. The broad definition for the MeSH term “Deprescriptions” may be an obstacle for its use in the deprescribing field.

The “objective” approach led to the identification of terms that could have been missed with a mor “subjective” approach. The term frequency analysis is the key concept of the “objective” approach. Our result suggests the term frequency analysis appears to be useful when literature is rare and relatively new, as with deprescribing.

We combined relevant terms using an incremental process [19], to insure a better reproductivity. Various processes have been used in the search filter literature. Some authors used statistical regression to combine relevant terms [23], others used automated computer process [10]. To our knowledge, no guidelines are yet available for building a filter from relevant terms. Further research is needed to explore the most efficient process.

### Limitations

Our study has some limitations. First, we identified less relevant articles than expected. We used the randomization ratio from Hausner et al. to split the reference set [16]. This led to a smaller validation group with less relevant articles than expected and a larger 95% CI for sensitivity. Our preliminary estimation of 2% prevalence of deprescribing articles was optimistic, since the prevalence in our reference set was 0.94%. Second, estimated precision and specificity should be interpreted with caution due to the high number of articles retrieved in each database. Lefebvre et al. reported that precision of searches undertaken in systematic review is often lower to 2%. We obtained 8% precision with our filters, but this estimate is likely influenced by the journals included in the reference set. We selected journals that published the most on deprescribing and that are thus not representative of the entire Medline and Embase databases. This approach was necessary to identify a high number of relevant articles, as deprescribing literature is dispersed. Some terms identified as specific to deprescribing in our reference set are more frequently used in other biomedical fields. Iansavichus et al. avoided this issue by randomly selecting journals in targeted database [10]. However, such process would have represented a substantial screening workload that was incompatible with our time and resources constraints. We believe a complementary pragmatic approach could overcome the substantial workload of the screening process. This pragmatic approach would prospectively compare search strategies used by research teams working on deprescribing reviews. The compared search strategies would use either the deprescribing filters we developed, or deprescribing terms identified independently by research teams. Articles retrieved with both strategies would be compared, and performances of the search filters would be calculated accordingly. This process would allow an external validation of both sensitivity and precision of the filters. Third, we used a validated so-called “objective” methodology [24], but subjectivity still remained in the process. Choice of pertinent terms or phrases for deprescribing was still partly dependent on the team’s expertise and thus may question reproducibility. Finally, effectiveness of the filters used in a search strategy designed for a specific research question in an actual systematic review remain uncertain. Further studies are necessary to assess the effectiveness and time saving of the filters when used in real-life systematic review search strategies.

## Conclusion

We developed two search filters to identify deprescribing articles in Medline via PubMed and Embase via Embase.com, with a sensitivity of 92% (95% CI: 83–97) and 91% (95% CI: 82–96) respectively.

These filters are intended to be used as part of search strategies designed for literature reviews on deprescribing-related questions. We recommend researchers to add specific medication or population terms to focus the search on their research question and improve performance. Further research is needed to evaluate the effectiveness and efficiency of the filters in a systematic review search strategy.

## Supplementary Information

**Additional file 1.**

## Data Availability

The datasets used and analyzed during the current study are available from the corresponding author on reasonable request.
