# Supplementary material for: Development and validation of search filters to identify articles on deprescribing in Medline and Embase
Source: BMC Med Res Methodol. 2022 Mar 25;22:79. doi: 10.1186/s12874-022-01515-x (PMC8953136; doi:10.1186/s12874-022-01515-x)
Supplement: Supplementary file 1 — Additional file 1. [file 12874_2022_1515_MOESM1_ESM.docx]

**Development of search filters to identify articles on deprescribing in Medline via PubMed and Embase**

Thomas Morel^a^, MSc, ORCID: 0000-0003-2141-1442

Jérôme Nguyen-Soenen^a^, MD, MSc, ORCID: 0000-0002-6023-8892

Wade Thompson^b,c^, PharmD, PhD, ORCID: 0000-0002-8268-4092

Jean-Pascal Fournier^a^, MD, PhD, ORCID : 0000-0002-9971-0672

^a^Département de Médecine Générale, Université de Nantes, France

^b^Women's College Hospital Research Institute, Toronto, Ontario, Canada

^c^Research Unit of General Practice, University of Southern Denmark, Odense, Denmark

**Reprints and correspondence:** Dr Jean-Pascal Fournier, Département de Médecine Générale, Faculté de Médecine, Université de Nantes, France. Tel: 33 (0) 24 041 1129, Fax: 33 (0) 24 041 2879, e-mail: [jean-pascal.fournier@univ-nantes.fr](mailto:jean-pascal.fournier@univ-nantes.fr)

**SUPPLEMENTARY MATERIALS**

**Supplementary Table S1.** Search terms identified for PubMed

| **Search term** | **Field searched** | **Retrieved relevant records in the development set (n=149)** | **Retrieved non relevant records in the development set (n=15,678)** | **Sensitivity in the development set (%)** | **Number of records identified in Embase^*^** |
| --- | --- | --- | --- | --- | --- |
| Inappropriate* | tiab | 68 | 367 | 45.6 | 72,029 |
| inappropriate | tiab | 67 | 367 | 45.0 | 64,169 |
| deprescri* | tiab | 65 | 25 | 43.6 | 1080 |
| Reduc* AND medication* | tiab | 65 | 391 | 43.6 | 77,310 |
| Deprescrib* | tiab | 64 | 24 | 43.0 | 1005 |
| Deprescribing | tiab | 64 | 23 | 43.0 | 975 |
| Medication* AND inappropriate | tiab | 61 | 215 | 40.9 | 5461 |
| Inappropriate prescribing | Mesh | 58 | 269 | 39.0 | 3731 |
| Review* AND medication | tiab | 50 | 409 | 33.6 | 40,358 |
| Reduc* AND inappropriate | tiab | 49 | 92 | 32.9 | 11,964 |
| Polypharmacy | tiab | 48 | 293 | 32.2 | 8792 |
| Deprescriptions | Mesh | 48 | 21 | 32.2 | 619 |
| Polypharmacy | Mesh | 42 | 250 | 28.2 | 5450 |
| Drug AND inappropriate | tiab | 41 | 197 | 27.6 | 6912 |
| Potentially inappropriate | tiab | 39 | 170 | 26.2 | 2198 |
| Inappropriate medication* | tiab | 35 | 126 | 23.5 | 1824 |
| Discontinu* | tiab | 32 | 233 | 21.5 | 134,702 |
| Reduc* AND polypharmacy | tiab | 31 | 65 | 20.8 | 2052 |
| Withdraw AND medication* | tiab | 30 | 17 | 20.1 | 8,215 |
| Medication review* | tiab | 29 | 130 | 19.5 | 1864 |
| reduc* AND prescribing | tiab | 28 | 190 | 18.8 | 10,558 |
| reducing | tiab | 27 | 369 | 18.1 | 452,982 |
| Drug-Related Side Effects and Adverse Reactions | Mesh | 26 | 415 | 17.4 | 33,728 |
| Inappropriate medications | tiab | 24 | 88 | 16.1 | 1094 |
| Discontinu* AND medication* | tiab | 23 | 71 | 15.4 | 14,537 |
| Potentially inappropriate medication* | tiab | 23 | 109 | 15.4 | 1273 |
| withdraw* | tiab | 22 | 122 | 14.8 | 132,083 |
| withdrawal | tiab | 21 | 89 | 14.1 | 95,296 |
| Medication review | tiab | 20 | 112 | 13.4 | 1490 |
| discontinuation | tiab | 20 | 148 | 13.4 | 54,062 |
| Discontinuat* | tiab | 20 | 160 | 13.4 | 56,145 |
| Potentially inappropriate medications | tiab | 19 | 75 | 12.7 | 866 |
| Inappropriate prescribing | tiab | 19 | 98 | 12.7 | 1572 |
| Medication reviews | tiab | 18 | 47 | 12.1 | 674 |
| Medication use | tiab | 17 | 220 | 11.4 | 17,967 |
| Appropriateness | tiab | 16 | 125 | 10.7 | 22,320 |
| Drug reactions | tiab | 15 | 293 | 10.1 | 15,361 |
| Adverse drug reactions | tiab | 15 | 287 | 10.1 | 13,362 |

^*^ On June 16, 2021

**Supplementary table S2.** Search terms identified for Embase

| **Search term** | **Field searched** | **Retrieved relevant records in the development set (n=149)** | **Retrieved non relevant records in the development set (n=15,678)** | **Sensitivity in the development set (%)** | **Number of records identified in Embase^*^** |
| --- | --- | --- | --- | --- | --- |
| deprescrib* | tiab | 63 | 19 | 42.3 | 1414 |
| deprescri* | tiab | 63 | 19 | 42.3 | 1495 |
| inappropriate | tiab | 62 | 345 | 41.6 | 90,969 |
| Inappropriate* | tiab | 62 | 367 | 41.6 | 102,374 |
| deprescribing | tiab | 60 | 18 | 40.3 | 1349 |
| Polypharmacy | Emtree | 45 | 374 | 30.2 | 18,586 |
| Reduc* near/5 medication* | tiab | 38 | 100 | 25.5 | 19,213 |
| Potentially inappropriate | tiab | 37 | 165 | 24.8 | 3459 |
| polypharmacy | tiab | 37 | 262 | 24.8 | 13,968 |
| Medication* near/4 inappropriate | tiab | 36 | 125 | 24.2 | 3720 |
| Review* near/5 medication | tiab | 33 | 195 | 22.1 | 7680 |
| Discontinu* | tiab | 32 | 232 | 21.5 | 220,736 |
| Inappropriate medication* | tiab | 31 | 118 | 20.8 | 2890 |
| Medication review* | tiab | 28 | 118 | 18.8 | 3788 |
| reducing | tiab | 27 | 369 | 18.1 | 592,417 |
| Drug withdrawal | Emtree | 27 | 414 | 18.1 | 213,198 |
| Deprescription | Emtree | 26 | 5 | 17.4 | 626 |
| Potentially inappropriate medication | Emtree | 24 | 122 | 16.1 | 1856 |
| Inappropriate medications | tiab | 23 | 81 | 15.4 | 1094 |
| Potentially inappropriate medication* | tiab | 22 | 105 | 14.8 | 1965 |
| Medication therapy management | Emtree | 22 | 195 | 14.8 | 12,417 |
| Withdraw* | tiab | 21 | 120 | 14.1 | 185,700 |
| Withdrawal | tiab | 20 | 88 | 13.4 | 133,725 |
| discontinuation | tiab | 20 | 147 | 13.4 | 96,255 |
| Discontinua* | tiab | 20 | 159 | 13.4 | 102,811 |
| Inappropriate prescribing | Emtree | 19 | 163 | 12.8 | 4791 |
| Medication reviews | tiab | 18 | 47 | 12.1 | 674 |
| Potentially inappropriate medications | tiab | 18 | 71 | 12.1 | 866 |
| Medication review | tiab | 18 | 94 | 12.1 | 1490 |
| Medication use | tiab | 17 | 217 | 11.4 | 30,702 |
| Reduc* near/5 prescribing | tiab | 16 | 54 | 10.7 | 3335 |
| Appropriateness | tiab | 16 | 122 | 10.7 | 31,630 |
| Inappropriate prescribing | tiab | 15 | 72 | 10.1 | 2082 |

^*^ On June 16, 2021

**Supplementary Material 1 -** Study Protocol

**Development and evaluation of a deprescribing topic search filter with maximized sensitivity on MEDLINE and Embase**

**INTRODUCTION**

Polypharmacy is a major challenge in primary care, as its prevalence has greatly increased over the last 30 years (1). Although its impact on patients' health status in general remains uncertain, a meta-analysis has highlighted a direct positive association between polypharmacy and mortality (2). Within the problem of polypharmacy lies the problem of inappropriate medications, especially in elderly patients. A study of polypharmacy using the START/STOPP criteria (3) found a direct association between polypharmacy and inappropriate medication (4). As this elderly population is particularly fragile, there is a need for interventions aiming at reducing the burden of polypharmacy.

It was in this context that the concept of deprescribing has been elaborated in recent years. Deprescribing can be defined as a “process of withdrawal of an inappropriate medication, supervised by a health care professional with the goal of managing polypharmacy and improving outcomes” (5). Beyond this definition, the concept of deprescribing refers to a complex process part of the "good prescribing continuum", in which the health professional identifies all the patient's medications, measures their potential adverse effects, and precisely monitors the withdrawal or the reduction of a medication by controlling the improvement of an outcome that the patient values (6).

The multiplication of deprescribing publications represents a challenge for the elaboration of recommendations, since different types of research are indexed under this term (7). For example, the study of discontinuation, *i.e.* the study of the outcomes related to the stopping of the medication (whether supervised or not, and whether aiming at improving patients outcomes or not) is often indexed as deprescribing, although it does not meet the definition of deprescribing. However, the study of discontinuation is essential and provides evidence which then allows the development of complex deprescribing interventions, in which it is the intervention itself that is evaluated, and the cessation of treatment might only be a secondary outcome for the evaluation of this intervention, as Gnjidic *et al.* explain (7).

In order to carry out a systematic review on deprescribing interventions, it is recommended to use carefully designed search strategies that would capture all relevant references. Specific recommendations in the development of these search strategies have been developed by the Cochrane Collaboration (8). Among these strategies, search filters can be particularly useful to elaborate the final search strategy while saving significant time for researchers. The use of such search filters is possible assuming that their development and performance have been properly assessed. Recommendations for the development of search filters have recently been proposed (9). The validation of a filter must be performed against a carefully developed "Gold Standard". For the selection of terms to be used in search strategies, the conceptual approach is usually preferred (8). However, a more objective approach via text mining is emerging. Hausner *et al.* have developed a detailed objective strategy that can be adapted to the creation of a search filter (10).

Information specialists have developed numerous methodological search filters that are currently used as references, in particular to identify randomized controlled trials (11). However, relatively few topic search filters have been developed (12), and none to our knowledge have been created for deprescribing studies. Similarly, their is currently no reference set of publications that could be used as “Gold Standard” for the creation and development of a search filter exists to our knowledge.

Doudet-Bouget *et al.* created four preliminary filters using the relative recall methods, but these filters were limited by the imprecise selection of the studies included in the reference set. (13)

Thus, the aim of our study is to create a reference set of deprescribing studies by handsearching the literature, and use it to develop and validate a specific deprescribing topic search filter, using an objective strategy adapted from Hausner *et al.*

**METHODS**

**Study design**

The aim of this study is to create and validate a search filter, by analogy with the validation of a diagnostic test. The reference set can be defined in this study by the manual review of a defined sample of the literature. This set can be considered a Gold Standard.

**Creation of the reference set**

The first step in developing a deprescribing filter is to create a reference set of references that allows to both develop and validate the filter. To our knowledge, there is no deprescribing study register that could be used for this purpose. Thus, we will create the reference set through a manual review of the literature.

We therefore selected eight journals (appendix 1) for their scope (general internal medicine, pharmacology and geriatrics), their high number of references on deprescribing, and on the basis of the experience deprescribing international experts. We chose of a ten-year back-up period, even though deprescribing have only defined in 2015. This time frame choice has been motivated on the fact that studies on deprescribing procedures may have been published before this definition has been developed and being indexed using specific keywords.

All references published by these journals and indexed in MEDLINE from 01/01/2011 to 31/12/2020 will be extracted. All titles and abstracts will be included in the RYYANN software (14). If no abstract is available, the full text will be obtained. The references will be reviewed independently by two reviewers for each one of them. Four reviewers will participate in the process. They will classify references in three categories: « deprescribing references », « other » and « uncertain ». When all these references have been screened, the full texts of the references classified as “uncertain” will be retrieved, and their classification in « deprescribing references » or « other » will be resolved by discussion. If the disagreement persists, a third researcher will be included in the discussion to reach consensus.

The agreement between the reviewers will be verified by calculating a kappa score on all references reviewed. The agreement will be considered acceptable if the kappa coefficient is greater than 0.8.

After classification, references will be randomly divided into two subsets: i) the development set, which will be used for the development procedure of the filter, and ii) the validation set, which will be used for the assessment of the performance of the filter. The references included in the validation set will be used to validate the sensitivity, specificity and accuracy of the filter, in the manner of a diagnostic study (Table 1).

**References selection**

Several inclusions and exclusions criteria have been established through discussions with experts in the field. The aim of these criteria is to include only references that meet the definition of deprescribing and therefore to exclude articles on discontinuation only. Since the objective is to create a topic search filter, all deprescribing references will be included, regardless of their methods and/or types of reports (original research study, systematic reviews, commentaries, editorials).

The criteria for inclusion will match the following definition: references relevant to deprescribing defined as intervention designed to favorize the cessation or the dose reduction of an inappropriate [1] long-term medication [2], supervised by a health care professional [3] or according to a protocol design by a health care team [4].

Key criteria of inclusion:

Definition of concept

- 1. Inappropriate: defined as risk outweighing benefits
  2. Long term medication: defined by more than 4 weeks of use
  3. Supervised by health care professional OR
  4. following a health care team protocol: e.g. interactive mobile application to guide deprescription

Exclusion criteria that will be applied:

1. Studies that focuses on misused medication
2. Studies which only assesses the effects of discontinuation of medication (positive or negative effect), without deprescribing intervention.
3. Studies that evaluate patient-initiated discontinuation, that are not supervised by professional (adherence issue)

**Development of the search filter**

The analysis of the frequency of free terms in titles and extracts from the development set will be examined using the "Text Mining Package" of the open-source statistical software R. All the terms analyzed will be included in a tabular spreadsheet, and ranked in order of frequency, reflecting their sensitivity (*i.e.* their ability to identify the references of the development set). Only terms with a sensitivity > 20% will be retained (15). These terms will be referred to as “potentially eligible terms” (PET). The individual frequency of each of the PET will then be evaluated among the control references of the development set, and their sensitivity will be estimated. PET with a sensitivity of 2% or less among the controls will be selected. These terms will constitute the “candidate terms”.

All of these candidate terms will then be called up one by one in the Antconc software (16), to identify if and where a truncation needs to be added, and if particularly frequent word combinations can be identified. These word combinations must in themselves be related to the deprescribing definition.

Then, a specific individual search line in each database studied will be generated for each candidate term, taking into account the truncations and combinations of term identified.

The same procedure will be followed for MeSH terms, using the open source software PubReminer.

Finally, one filter per database will be constructed by adding the individual term lines in a trial-and-error process aimed at maximizing the sensitivity of the filter, while preserving its accuracy.

**Validation of the search filter**

*Validation against the validation set*

For each filter (database specific), the validation will be performed on the basis of the validation of a diagnostic test, using the validation set. Assuming a sensitivity of 90%, with an alpha error of 5% and a precision of 5%, the number of "deprescribing references" in the validation set would be of 139.

Each database will be queried by the specifically designed filter, and it will be checked which proportion of "deprescribing references" from the validation set is found and which proportion of “other" references is found. The results will be presented in a contingency table as shown in Table 1. The sensitivity (a/(a+c)) and specificity (d/(d+b)) of each filter will then be calculated.

An estimate of the filter accuracy will be calculated by taking the total number of “deprescribing references” found (validation set and development set together) in ratio to the total number of database references found ((a+e)/i). Accuracy can only be estimated since our reference set will not contain all the deprescribing studies of each database, but only those included in the 8 journals reviewed. Nevertheless, assuming that the prevalence of deprescribing studies is very low in each database, this estimated accuracy will be relatively close to the actual accuracy of each filter. In addition, the accuracy calculated in this case is underestimated compared to the actual accuracy of the filter.

*Testing the external validity of the filter*

These filters specific to each database will be developed and validated with a Gold Standard consisting of references systematically included in both databases. This can lead to a potential selection bias if the newspapers that publish on this subject and that are indexed in only one of the two databases have different characteristics than those included in both. This potential bias could overestimate the actual sensitivity of the filters, and thus reduce their external validity. To assess this possibility, a sensitivity analysis will be carried out for each of the databases.

Two journals each indexed in either MEDLINE or EMBASE, and which regularly publish on the subject have been identified (*Research in Social and Administrative Pharmacy* [MEDLINE], *Therapeutic Advances in Drug Safety* [EMBASE]). All references from 2019 from these two journals will be extracted and classified as "Deprescribing References" and "Other" by two independent authors. This year has been chosen since a preliminary analysis has shown that these journals have published several deprescribing studies during 2019. Then the performance of each filter will be tested by querying the database for the year of interest, and the sensitivity and specificity will then be estimated.

All analyzes will be conducted with R version 3.4.3.

**Ethics, reporting**

Ethical approval is not required for this study, given the nature of the data. The study results would be reported following the items of the "Standards for Reporting Diagnostic accuracy studies" (STARD) 2015.

**REFERENCES**

1. Wastesson JW, Morin L, Tan ECK, Johnell K. An update on the clinical consequences of polypharmacy in older adults: a narrative review. Expert Opin Drug Saf. 2018;17(12):1185‑96.

2. Leelakanok N, Holcombe AL, Lund BC, Gu X, Schweizer ML. Association between polypharmacy and death: A systematic review and meta-analysis. J Am Pharm Assoc JAPhA. déc 2017;57(6):729-738.e10.

3. O’Mahony D, O’Sullivan D, Byrne S, O’Connor MN, Ryan C, Gallagher P. STOPP/START criteria for potentially inappropriate prescribing in older people: version 2. Age Ageing. mars 2015;44(2):213‑8.

4. Galvin R, Moriarty F, Cousins G, Cahir C, Motterlini N, Bradley M, et al. Prevalence of potentially inappropriate prescribing and prescribing omissions in older Irish adults: findings from The Irish LongituDinal Study on Ageing study (TILDA). Eur J Clin Pharmacol. mai 2014;70(5):599‑606.

5. Reeve E, Gnjidic D, Long J, Hilmer S. A systematic review of the emerging definition of ‘deprescribing’ with network analysis: implications for future research and clinical practice. Br J Clin Pharmacol. 2015;80(6):1254‑68.

6. Scott IA, Hilmer SN, Reeve E, Potter K, Le Couteur D, Rigby D, et al. Reducing inappropriate polypharmacy: the process of deprescribing. JAMA Intern Med. mai 2015;175(5):827‑34.

7. Gnjidic D, Reeve E. Deprescribing: What do we know, and where to next? Br J Clin Pharmacol. 26 août 2020;

8. Lefebvre C, Glanville J, Briscoe S, Littlewood A, Marshall C, Metzendorf M-I, Noel-Storr A, Rader T, Shokraneh F, Thomas J, Wieland LS. Chapter 4: Searching for and selecting studies. In: Higgins JPT, Thomas J, Chandler J, Cumpston M, Li T, Page MJ, Welch VA (editors). Cochrane Handbook for Systematic Reviews of Interventions version 6.1 (updated September 2020). Cochrane, 2020. Available from www.training.cochrane.org/handbook. In.

9. Lefebvre C, Glanville J, Beale S, Boachie C, Duffy S, Fraser C, et al. Assessing the performance of methodological search filters to improve the efficiency of evidence information retrieval: five literature reviews and a qualitative study. Health Technol Assess Winch Engl. 2017;21(69):1‑148.

10. Hausner E, Guddat C, Hermanns T, Lampert U, Waffenschmidt S. Development of search strategies for systematic reviews: validation showed the noninferiority of the objective approach. J Clin Epidemiol. févr 2015;68(2):191‑9.

11. Glanville J, Kotas E, Featherstone R, Dooley G. Which are the most sensitive search filters to identify randomized controlled trials in MEDLINE? J Med Libr Assoc JMLA. 1 oct 2020;108(4):556‑63.

12. Damarell RA, May N, Hammond S, Sladek RM, Tieman JJ. Topic search filters: a systematic scoping review. Health Inf Libr J. mars 2019;36(1):4‑40.

13. Doudet-Bouget L. Développement de filtres de recherche pour identifier les articles traitant de la déprescription médicamenteuse dans Pubmed en utilisant la méthode du rappel relatif [Thèse d’exercice]. [France]: Université de Nantes. Unité de Formation et de Recherche de Médecine et des Techniques Médicales; 2019.

14. Mourad Ouzzani, Hossam Hammady, Zbys Fedorowicz, and Ahmed Elmagarmid. Rayyan — a web and mobile app for systematic reviews. Systematic Reviews (2016) 5:210, DOI: 10.1186/s13643-016-0384-4.

15. Hausner E, Waffenschmidt S, Kaiser T, Simon M. Routine development of objectively derived search strategies. Syst Rev. 29 févr 2012;1:19.

16. Anthony, L. (2020). AntConc (Version 3.5.9) [Computer Software]. Tokyo, Japan: Waseda University. Available from https://www.laurenceanthony.net/software.

**TABLES**

**Table 1:** Contingency table

|  | | Validation set |  | Development Set |  | Database |
| --- | --- | --- | --- | --- | --- | --- |
|  |  | Deprescribing References | Other | Deprescribing References | Other |  |
| Objectiv search filter | References retrieved | a | b | e | f | i |
|  | References not retrieved | c | d | g | h |  |

**APPENDIX**

**Appendix 1 -** Journals that will be included in the hand-searching

*European Journal of Clinical Pharmacology*

*British Journal of Clinical Pharmacology*

*International Journal of Clinical Pharmacy*

*Drugs & Aging*

*Age and Ageing*

*Journal of the American Geriatrics Society*

*British Journal of General Practice*

*JAMA Internal Medecine*

**Supplementary Material 2-** UK InterTASC Information Specialists’Sub-Group (ISSG) Search Filter Appraisal Checklist

1. **Information**

**A.1. State the author’s objective.**

To developed and validate search filters to retrieved deprescribing articles

**A.2. State the focus of the research.**

Sentitivity-maximizing search filter

**A.3. Database(s) and search interface(s).**

Embase with Embase.com and Medline with PubMed

**A.4. Describe the methodological focus of the filter.**

Topic search filter, no methodological focus

**A.5. Describe any other topic that forms an additional focus of the filter (e.g., clinical topics such as breast cancer, geographic location such as Asia, or population grouping such as paediatrics).**

Topic filters focus on deprescribing literature

**A.6. Other information**

Not applicable

1. **Identification of a gold standard (GS) of known relevant records**

**B.1. Did the authors identify 1 or more gold standards (GSs)?**

One GS developed for the study: articles from eight journals indexed both in Medline and Embase published between 2011 and 2020. The GS was then randomly divided in a development set (70%) and a validation set (30%). Randomization was stratified on articles relevance.

**B.2. How did the authors identify the records in each GS?**

All records from the GS were independently screened in duplicate by three researchers.

**B.3. Report the dates of the records in each GS.**

2011 to 2020

**B.4. What are the inclusion criteria for each GS?**

Articles were included regardless to the population studied and the control and the outcome used.

Inclusion criteria used for deprescribing intervention was:

1_ the intervention was designed with the goal of stopping or reducing the dose of an

2_ inappropriate medication

3_ inappropriate medications are long-term medication

4_ the intervention was supervised by a health care professional

5_ or the intervention was performed according to a protocol designed by a health care team.

**B.5. Describe the size of each GS and the authors’ justification, if provided (e.g. the size of the GS may have been determined by a power calculation**).

The GS included 23.792 articles. A power calculation was performed to determine the minimal necessary size to validate a 90% sensitivity with a confidence intervalof 95% +/- 5% and a estimated prevalence of 2% for deprescribing articles.

**B.6. Are there limitations to the gold standard(s)?**

Journals were known to have regularly published on deprescribing literature. They are thus not representative of the entire Medline and Embase databases.

**B.7. How was each GS used?**

Development set:

To identify potential search terms

To derive potential strategies (groups of terms)

To test internal validity

Validation set:

To test external validity

**B.8. Other observations.**

Not applicable

1. **How did the researchers to identify the search terms in their filter(s)?**

**C.1. Adapted a published search strategy.**

No

**C.2. Asked experts for suggestions of relevant terms.**

No

**C.3. Used a database thesaurus.**

No

**C.4. Performed statistical analysis of terms in a GS set of records (see B above).**

Yes, statistical analysis was performed to retrieved free-text terms, truncated terms or phrases used in titles/abstracts and subject headings used for each article. Terms were selected if they were identified in 10% or more of relevant articles and 3% or less in non-relevant articles. Generic terms were excluded.

**C.5. Extracted terms from the GS of records (see B above).**

Yes, with a statistical analysis.

**C.6. Extracted terms from some relevant records (but no a GS)**

No

**C.7. Tick all types of search terms tested.**

Subject headings: yes

Text words (e.g. in title and abstract): yes

Publication types: no

Subheadings: no

Check tags: no

Other: no

**C.8. Include the citation of any adapted strategies.**

No

**C.9. How were the (final) combination(s) of search terms selected?**

Search terms selected were combined until no more selected terms could increase sensitivity, in maximizing-sensitivity process.

**C.10. Were the search terms combined (using Boolean logic) in a way that is likely to retrieve the studies of interest?**

**C.11. Other observations.**

Not applicable

1. **Internal validity testing (This type of testing is possible when the search filter terms were developed from a known GS set of records.)**

**D.1. How many filters were tested for internal validity?**

Internal validitity was test against the development set.

Two filters were tested: the Medline deprecribing search filter (Mdsf) and the Embase deprescribing search filter (Edsf). Interna validity testing was perfomed in the development set.

Both filters were the final and most sensitive combination of search terms selected.

**D.2. Was the performance of the search filter tested on the GS from which it was derived?**

Mdsf : Yes, internal validity was assessed on the development set.

Edsf : Yes, internal validity was assessed on the development set.

**D.3. Report sensitivity data (a single value, a range, “Unclear” or “Not reported”, as appropriate).**

Mdsf: 96%

Edsf: 97%

**D.4. Report precision data (a single value, a range, “Unclear” or “Not reported”, as appropriate).**

Mdsf: 8%

Edsf: 9%

**D.5. Report specificity data (a single value, a range, “Unclear” or “Not reported”, as appropriate).**

Mdsf: not reported

Edsf: not reported

Available on request to the authors

**D.6. Other performances measures reported**

Mdsf: no

Edsf: no

**D.7. Other observations.**

Not applicable.

1. **External validity testing (This section relates to testing the search filter on records that are different from the records used to identify the search terms.)**

**E.1. How many filters were tested for external validity on records different from those used to identify the search terms?**

External validity was test against the validation set.

Two filters were tested: the Medline deprecribing search filter (Mdsf) and the Embase deprescribing search filter (Edsf).

**E.2. Describe the validation set(s) of records, including the interface**

The validation set was a random part of 30% of the initial GS. Articles included in the validation set was not include in the development set. Validation set was accessed with both PubMed and Embase.com interface.

**E.3.  On which validation set(s) was the filter tested?**

Both Mdsf and Edsf was tested on the same validation set

**E.4.  Report sensitivity data for each validation set (a single value, a range, ‘‘Unclear,’’ or ‘‘Not reported,’’ as appropriate).**

Mdsf: 92% (95% CI: 83-97)

Edsf: 91% (95% CI: 82-96%)

**E.5.  Report precision data for each validation set (report a single value, a range, ‘‘Unclear,’’ or ‘‘Not reported,’’ as appropriate).**

Mdsf: 8% (95% CI: 6-10)

Edsf: 8% (95% CI: 6-10)

**E.6.  Report specificity data for each validation set (a single value, a range, ‘‘Unclear,’’ or ‘‘Not reported,’’ as appropriate).**

Mdsf: 90% (95% CI: 89-91)

Edsf: 90% (95% CI: 89-91)

**E.7.  Other performance measures reported.**

Sensitivity to identify deprescribing original articles

Mdsf : 97% (95% CI: 85-100)

Edsf: 86% (95% CI: 71-95)

**E.8.  Other observations.**

Not applicable

1. **Limitations and comparisons**

**F.1.  Did the authors discuss any limitations to their research?**

Yes, see Discussion and Limitation in the paper

**F.2.  Are there other potential limitations to this research that you have noticed?**

Not applicable

**F.3.  Report any comparisons of the performance of the filter against other relevant published filters (sensitivity, precision, specificity, or other measures).**

Not applicable

**F.4.  Include the citations of any compared filters.**

Not applicable

**F.5.  Other observations and/or comments.**

Not applicable

1. **Other comments (This section can be used to provide any other comments. Selected prompts for issues to bear in mind are givent below.)**

**G.1. Have you noticed any errors in the document that might impact on the usability of the filter?**

Not applicable

**G.2. Are there any published errata or comments (e.g. in the MEDLINE records)?**

Not applicable

**G.3. Is there public access to prepublication history and/or correspondence?**

Not applicable

**G.4. Are further data available on a linked site or from the authors?**

Data are available on reasonable request to the corresponding author

**G.5. Include references to related papers and/or other relevant materials.**

Not applicable

**G.6. Other comments.**

Not applicable

**Supplementary Material 3 -** List of deprescribing experts

Dr Barbara FARRELL, BScPHM, PharmD, RPh, ACPR, FCSHP

Senior Investigator

Bruyère Research Institute, University of Ottawa

Dr Emily REEVE, PharmD, PhD.

Senior Research Fellow, Senior Lecturer of Pharmacy Practice

UniSA & Health Sciences, University of South Australia

Dr Wade Thompson, PharmD, PhD.

Postdoctoral Researcher

Women’s College Hospital
